# Predicting protein disorder by analyzing amino acid sequence

**DOI:** 10.1186/1471-2164-9-S2-S8

**Published:** 2008-09-16

**Authors:** Jack Y Yang, Mary Qu Yang

**Affiliations:** 1 Harvard Medical School, Harvard University, P.O. Box 400888 Cambridge, MA 02115, USA; 2 National Human Genome Research Institute, National Institutes of Health, Bethesda, MD 20852, USA

## Abstract

**Background:**

Many protein regions and some entire proteins have no definite tertiary structure, presenting instead as dynamic, disorder ensembles under different physiochemical circumstances. These proteins and regions are known as Intrinsically Unstructured Proteins (IUP). IUP have been associated with a wide range of protein functions, along with roles in diseases characterized by protein misfolding and aggregation.

**Results:**

Identifying IUP is important task in structural and functional genomics. We exact useful features from sequences and develop machine learning algorithms for the above task. We compare our IUP predictor with PONDRs (mainly neural-network-based predictors), disEMBL (also based on neural networks) and Globplot (based on disorder propensity).

**Conclusion:**

We find that augmenting features derived from physiochemical properties of amino acids (such as hydrophobicity, complexity etc.) and using ensemble method proved beneficial. The IUP predictor is a viable alternative software tool for identifying IUP protein regions and proteins.

## Background

Proteins are composed of one or more chains of amino acids, and exhibit several levels of structure. The primary structure is defined by the sequence of amino acids comprising each chain, while the secondary structure is defined by local, repetitive spatial arrangements, which falls into three basic categories: helix, strand, and coil. The tertiary structure is defined by how the chain folds into a three-dimensional configuration, while the quaternary structure is concerned with how different chains combine into multisubunit or oligomeric, protein (protein complexes). Most proteins function only when folded into a particular configuration. Recently, a class of proteins has been discovered that do not fold into any particular configuration – instead of folding into specific 3-D structures, they exist as dynamic ensembles in their native state. These proteins have been variously called natively unfolded, natively disordered or Intrinsically Unstructured regions and Proteins (IUP) [1-7]. Unlike regular proteins, which unfold and lose their ability to function when subjected to environmental challenges such as detergents, urea, or heat, IUP may continue to function under such conditions, as they do not have to be folded into a particular configuration in order to carry out their function. An IUP protein usual does not involve with catalysis process that functioning as an enzyme, because catalysis requires tightest-binding to transition state, which binding specificity most likely requires structured active site, an IUP functions in signaling and have been associated with a wide range of protein functions such molecular recognition, molecular assembly and disassembly as well as protein modification. IUP regions also play a central role in diseases characterized by protein misfolding and aggregation [1-5]. Furthermore, the identification of such regions can aid both structure determination and sequence alignment, and may also aid in drug design. The identification of IUP regions from the primary structure of a protein is thus an important but difficult problem [1-5]. IUP can be identified through protein tertiary structure. Traditionally, the tertiary structure of proteins is determined using experimental methods such as X-ray crystallography, Overhauser Effect Enhanced Nuclear Magnetic Resonance spectroscopy (NMR), and Circular Dichroism Spectroscopy (CDR). However, these experimental methods are usually time consuming and often have their own limitations and problems. Since Dunker et. al. developed the first IUP predictor – PONDR [3], consequential development of IUP predicators includes disEMBL [6], and Globplot [7]. We had developed a number of IUP and membrane protein predictors [8-10] that use amino acid sequences as inputs and that give IUP and structured protein assignments as outputs. This paper is a continuation of the earlier IUP predictors we developed before [8-10]. Our predictors use protein primary structure information only and contain three parts: feature generation, classification and ensemble methods.

## Methods

### Feature generation from primary structure

The first step in constructing a classifier is to choose the features that the classier uses. Performance of a given classifier depends on the set of features that are used. Our feature extraction is based on the physiochemical analysis of protein sequences.

#### Features from compositions of peptide sequences

We studied the characteristic of amino acid residues in sequences. We found that different properties of amino acid in sequence tend to encode structural information. It appears different protein folding classes can be identified by the differences in their amino acid compositions. Detailed analysis of patterns of sequences leads us some important discoveries. Analyzing the peptide patterns in sequences have convinced us that different protein folding classes can be identified by the differences in their amino acid compositions. We conclude that distinguishing peptide patterns in sequences provides useful information to detect different protein classes and/or discover new classes of proteins.

The first set of 20 features is derived from first order statistics, regarding amino acid compositions in primary sequence. Let us define amino acid set as:

$\tilde{A}$ = {*A*, *C*, *D*, *E*, *F*, *G*, *H*, *I*, *K*, *L*, *M*, *N*, *P*, *Q*, *R*, *S*, *T*, *V*, *W*, *Y*}.

These twenty letters in the set $\tilde{A}$ represent the one-letter symbols for 20 amino acids. We use a window with length *L *centered at each amino acid residue to extract features. Let *x*(*j*) ∈ $\tilde{A}$, *j *is the *j*^*th *^position of the amino sequence of protein. Hence, *x*(*j*) represents the amino acid at position *j *of the protein sequence. Let *M *denote the length of protein sequence. Since the window size shrinks [6] at the N-terminal and C-terminal of protein sequences, we utilized *k*_0 _and *k *as parameters to make the window size adjustable at the terminals. *k*_0 _and *k *are defined as follows:

(1) *k *= *max *{1, *i *- (*L*-1)/2}

and

(2) *k*_0 _= *min *{*M*, *i *+ (*L*-1)/2}

Let *P*_*i *_(*a*) represents the probability of an amino acid residue, which is denoted by "a"(a ∈ $\tilde{A}$), inside the window centered at position *j*, then

(3) $$P_{i}(a)=\frac{1}{k-k_{0}+1}\sum_{j=k_{0}}^{k}\delta_{x(j)a}$$

where *k*_0 _and *k *are defined in Equation (1) and (2), a ∈ $\tilde{A}$:

We then constructed more features by second order statistics regarding the pattern of one amino acid followed by another amino acid in the window. Let's define Ψ*a*_*m*_*a*_*n *_represent the pattern of a pair of amino acids, that is, amino acid *a*_*m *_followed by amino acid a_n_. Both *a*_*m *_and *a*_*n *_belong to amino acid set $\tilde{A}$. Second order statistic features are calculated by the following equation:

(4) $$P_{i}(\Psi a_{m}a_{n})=\frac{1}{k-k_{0}+1}\sum_{j=k_{0}}^{j=k-1}\delta_{x(j)a_{m}}\delta_{x(j+1)a_{n}}$$

where *k*_0 _and *k *are defined in (1), (2), *a*_*m *_∈ $\tilde{A}$, *a*_*n *_∈ $\tilde{A}$

$$\delta_{x(j),a_{m}}\delta_{x(j+1),a_{n}}=\{\begin{array}{ll} 1 & \text{if amino acid x}(\text{j})={\text{a}}_{\text{m}}\text{ and x}(\text{j}+1)={\text{a}}_{\text{n}}\\ 0 & \text{otherwise} \end{array}$$

Next we introduce a 9-gram encoding scheme based on the physiochemical properties of amino acids. The 20 amino acid residues can be clustered into 9 groups as shown in Table 1. Then, we defined the 2-tuple code set as following:

**Table 1:** Encode scheme based on physiochemical properties of amino acids

Group	Residues	Description
g1	C	Highly conserved
g2	M	Hydrophobic
g3	N, Q	Amides, polar
		Acids, positive,
g4	D, E	polar
g5	S, T	Alcohols
g6	P, A, G	Aliphatic, small
g7	I, V, L	Aliphatic
g8	F, Y, W	Aromatic
g9	H, K, R	Bases, charged

Ω = {g1, g2, g3, g4, g5, g6, g7, g8, g9}, and g ∈ Ω

There are two advantages to this 9-gram encoding scheme. First, for small window sizes, there may not be sufficient data to accurately represent the first and second order statistics of 20 amino acids; this will be less of a problem with the 9-gram encoding because there are fewer first and second order statistics to estimate than when the 20 amino acid encoding is used. Second, the 9-gram scheme can reduce computational complexity if it is used as an alternative.

Modify equations (3) and (4) by the 9-gram encode scheme, we then calculate on the first order statistics and second order statistics of the 9-gram encoding scheme for each amino acid residue in a sequence using the same window accordingly.

We also generated features by using protein family profiles or position-specific scoring matrices from PSI-BLAST. For a sequence of length N, an N*20 family profile is constructed based on the multiple alignment of homologues found during the PSI-BLAST search. Feature *P*_*j*_(*a*) is the averaged log-odds of amino acid "*a*" in the neighbourhood of sequence position *j *as calculated below:

(5) $$p_{j}(a)=\frac{1}{k-k_{0}+1}\sum_{j=k_{0}}^{k}S_{x(j)}(a)$$

#### Sequence complexity

The complexity of the sequence is used as a features. According to the past research [1-5], Most likely IUP have low complexity patterns. *K*2-entropy is used to measure the local complexity of the amino acid sequence. The complex of each amino acid residue in the sequence is calculated in the same window as previous defined, which is given by:

(6) $$C_{i}=-\sum_{n=1}^{20}P_{i}(a_{n})\log_{2}P_{i}(a_{n})$$

where *a*_*n *_∈ $\tilde{A}$, and *P*_*i *_is calculated by Equation (3).

#### Hydrophobicity

Since hydropathy is an important determinant of protein-chain fold, calculation of hydropathy could be useful for IUP prediction. We therefore use relative hydrophobicity of each amino acid, called hydropathy in the feature space. The feature for hydropathy *H*(*i*) at position *i *is the average of hydropathy in the feature window for given hydropathy scale is calculated as following:

(7) $$H(i)=\frac{1}{k-k_{0}+1}\sum_{j=k_{0}}^{k}Hydropathy(j)$$

There are several different hydropathy scales generated by different methods. Equation (7) is used for generating features based on four different hydropathy scales. However, by comparing joint probability distributions of intrinsically unstructured and structured proteins for all different hydropathy scales, we found that Kytes-Doolittle's scale [11] is best in distinguishing IUP from structured proteins. This best feature can be also selected from principle component analysis as showing in the feature selection session.

#### IUP propensities

Finally, we also use amino acid IUP propensities [7], which is a scale to measure how likely an amino acid is to be unfolded. Comparisons on two sets of propensities are shown in Table 2. The average of IUP propensity associated with *j*^*th *^residue in the sequence given in a window with length *L*is calculated as below:

**Table 2:** Russell/Linding and Deleage/Roux: Disorder Propensities

Residue	Russell/Linding	Deleage/Roux
P	0.55232	1.117
G	0.43323	0.6675
N	0.22989	0.479
D	0.22763	0.4645
S	0.14288	0.2965
T	0.00887	0.145
H	-0.00121	0.135
C	-0.00151	-0.1255
K	-0.10001	-0.0495
R	-0.17659	-0.179
Q	-0.18768	-0.055
E	-0.20469	-0.2745
Y	-0.20751	0.0825
F	-0.22557	-0.497
M	-0.2259	-0.4765
W	-0.24338	-0.257
A	-0.26154	-0.275
L	-0.33793	-0.4385
V	-0.38618	-0.7055
I	-0.42224	-0.515

(8) $$R(j)=\frac{1}{k-k_{0}+1}\sum_{j=k_{0}}^{k}IUPpropen\;(j)$$

The *k *and *k*_*o *_are defined in (1), (2).

The selection on a better propensity feature is illustrated in the feature selection session using a distance gauge.

#### Feature selection for high-dimensional data

The above feature extraction step can generate 537 features for each amino acid residue in a sequence as summarized below:

• 20 from first order statistics of 20 amino acids;

• 400 from second order statistical of 20 amino acids;

• 9 from first order statistics of 2-tuple code;

• 81 from second order statistics of 2-tuple code;

• 20 Features based on PSI-BLAST Profiles;

• 1 from complexity;

• 4 from different hydropathy scales;

• 2 from different unstructured propensity scales.

High-dimensional data requires a feature selection step to address the curse-of-dimensionality. In addition, a large number of features often make the learning algorithm scale poorly [1]. Generally, feature selection strategies are either wrapper-based, or embedded, or filter-based.

#### Wrapper-based feature selection

Wrapper algorithms use the interactions between feature selection and the learning algorithm by involving the learning algorithm in the feature selection step. If they are not over-fitting and are not so expensive computationally, wrappers would be the best feature selection algorithms, since they also depend on the inductive principles of the learning algorithms.

#### Embedded feature selection algorithms

Decision Trees and CART (classification and regression trees) exemplify embedded feature selections; the process of selecting a feature to split at each node of the tree is implicitly a feature-selection step.

#### Filter-based feature selection

Filter-based feature selections select features before the data is passed to a learning algorithm. They are used as pre-processing steps to model selections and learning. Since these algorithms are independent of any learning algorithms and they are used in the preliminary steps in learning, their computational complexities are usually not high and are much faster than wrapper-based algorithms.

We studied several feature selection algorithms include information gain, T-test, the Chi-square goodness-of-fit test, the bi-normal separation (BNS), Fisher permutation test, and distance measurement.

We are especially in favour of distance measures because they can reduce computational complexity efficiently. Let's consider a two-class problem. Given two features: X and Y, D (X) and D (Y) measure the separation of two classes subject to feature X and feature Y, respectively. The distance measure D (X) for feature X is defined as follows:

(9) $$D(X)=\frac{m_{1}-m_{2}}{\sqrt{\sigma_{1}^{2}+\sigma_{1}^{2}}}$$

where *m*_1 _and *m*_2 _are the mean values of feature *X *for the class 1 and class 2, $\sigma_{1}^{2}$ and $\sigma_{2}^{2}$ are variance of feature *X *for the class 1 and class 2, respectively. The mean and variance for a given feature are calculated as follows:

(10) $$\begin{array}{c} m=\frac{1}{N}\sum_{i=1}^{N}x_{i}\\ \sigma^{2}=\frac{1}{N-1}\sum_{i=1}^{N}{\left(x_{i}-m\right)}^{2} \end{array}$$

where *x*_*i *_is the value of feature *X *for *i*^*th *^instance in one class, and *N *is the number of instances in that class. Modifying equations (10–11) gives us the calculation of *D*(*Y*) corresponding to feature. Now we obtain both *D*(*X*) and *D*(*Y*). If *D*(*X*) > *D*(*Y*), feature *X *is selected; otherwise feature *Y *is selected.

Distance measure can be in pair each time for every feature. After sorting these distances, we can select most important features for separating two classes. For example, for the feature on IUP propensities as showing in Tables 1, we choose Russell/Linding scale by the above decision rule. This also justify why they can build a single-feature simplest predictor.

From the above analysis, clearly, distance-measure based feature selection method works in a pair-wise manner. We are also in favour of another feature selection method called principle component analysis (PCA) to reduce the feature dimension.

Compare to previous approach, this particular feature selection scheme can handle multiple features simultaneously. PCA is also called Karhunen-Loeve (K-L) transformation. K-L transformation is an orthonormal transformation of a vector $\vec{X}$ to same dimensional vector $\vec{Y}$. In the transformation domain, the first principle component is the normalized linear combination with maximum variance; the second component has the next largest variance and so forth. Based on such ranking, only those with largest variance are preserved and the others are neglected. In fact the principle components are ranked by their ability to distinguish among classes. The implement [10] procedure is as follows. Assume there are N instances in the training set and M features, let $\vec{X}$ represent a population of N-dimensional vectors, mean value of each feature *m*_*x *_has been calculated. The mean value of each feature and KL transformation have been performed, the resulting covariance matrix of $\vec{Y}$ has been analyzed. According to the nature of those features, we divide them into four categories [10]: characterize of local compositions of amino acids, characterize patterns of local regions, characterize propensities of IUP, characterize physiochemical properties of local regions.

Evidently, there are four different hydropathy scales which result in four features regarding amino acid hydrophobicity property. These features are correlated with each other. The correlation factor *ρ*_*xy *_of two features X and Y can be obtained by the following equation:

(11) $$\rho_{xy}=\frac{E[XY]-E[X]E[Y]}{\sqrt{\left(E[X^{2}]-E{\left[X\right]}^{2}\right)}\sqrt{\left(E[Y^{2}]-E{\left[Y\right]}^{2}\right)}}$$

Among those four features, we choose the best one by using decision rule based on PCA. It turns out the Kyte-Doottle scale generate the best feature compared to the other three scales on IUP prediction [10].

After our feature selection procedure, we decide to use 59 selected features as input to our classifier. These features describe the amino acid compositions, hydropathy, complexity and IUP propensities from sequences. The experimental results show that these 59 features turn out to produce the best predicting accuracy for IUP classifier. The selected features are also cross checked with AAIndex  so that they are biologically significant.

### IUP predictor

We use the Recursive Maximum Contrast Tree Classifier (RMCT) we developed before [8-10]. This classifier utilizes K-Nearest Neighbour Classifier [12], where the nearest neighbours are defined by the tree structure [8-10,12]. After constructing the RMCT using the training data, the resulting tree can be used as a 2-class classifier. We define IUP residues as class 1, and structured protein peptide residues as class 0. K majority voting principle guides classification with RMCT on tree nodes. We calculate the distances between a test instance and instances in the training dataset by a distance measure *D*(*n*). We sort the distances in ascending order and get the first *K *minimum distances as our *K *decision-making instances in a tree node. Then the class label of the test instance is assigned according to the majority voting of class labels from these *K *decision-making distances.

**Figure 1:**
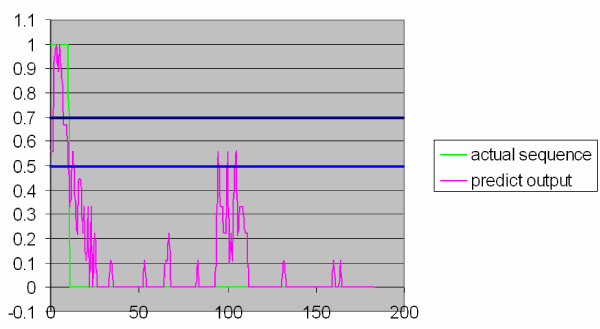
Example of IUP.

Detecting IUP is measured by the value *P*(*L *= 1) that is given by *K *majority voting rules based on the training instances and therefore is a real number between zero and one. AS shown in Figure 1 shows the IUP predictor interface, the y-axis on low-left window represents *P*(*L *= 1), while the x-axis represents the residue location (starting from number 1 to M – the length of sequence). *P *(*L *= 1) actually reflects a "probability" that a test instance is classified as class 1. If *P*(*L *= 1) is great than 1/2 by the *K *majority voting rule as described above, then this test instance should be classified as class 1, and if it is less than 1/2, it should be classified as class 0 (Default threshold value is set as 1/2). However such threshold can be adjustable in IUP classifier. The general rule for classification is that if *P*(*L *= 1) is greater than the threshold value (between 0 and 1), then the test instance is classified as class 1, otherwise it is classified as class 0. If we decrease the threshold value from 1/2, then more class 1 test instances can be detected. The trade off is that statistically, more class 0 instances are wrongly classified as class 1. Increasing the threshold has the effect of increasing the false negative rate and reducing the false positive rate. Varying threshold values is an essential technique in obtaining Receiver Operating Characteristic (ROC) curves. Once we set up a threshold value as the boundary, a test instance can be assigned a class label at each of the root, child and grandchild node (when N = 3) in RMCT classifier [8-10]. Finally classification decision (for N > 1) is made by the majority voting from the class labels of those nodes after each node has already made its own decision based on majority voting from the decision making instances in each node. Therefore, adjustable parameters in the IUP predictor are the window length L (used in generating features), the number of decision-making instances K in each node on RMCT and number of tree nodes participating in final classification decision (*N*).

### Ensemble methods

The performance of IUP predictor can be improved using ensemble methods; a diverse class of methods that seek to combine the decisions of several classifiers in order to improve classification accrues [12-15]. We exploited the implementation of combing boosting with bagging to improve prediction accuracy.

#### Combing Boosting with Bagging

We developed the Boosting with Bagging algorithm. Boosting is an algorithm that can be used to improve the performance of a classifier. While the original Boosting algorithm is due to Schapire [13], later Freund and Schapire introduced an improved algorithm called Adaboost, which was designed to handle 2-class classifiers. There were several extensions to the multi-class case, including Adaboost.M1 [14]. As we are interested in incorporating useful confidence information into IUP classifier, we combine bagging with a generalization of Adaboost.M1 called the CBoost algorithm [15] that allows confidence information to be incorporated. Our combined CBoost with bagging algorithm emphasizes on weaker learner for each boosting run.

Assuming we have *N *training instances, then we construct classify *f *(${\vec{x}}_{i}$). Class label *y*_*i *_is either 0 or 1. The square error of classify *f *(${\vec{x}}_{i}$) is given by each boosting round, The training set is denoted as T = {(*x*_*i*_, *y*_*i*_), *i *= 1,... *N*} where *x*_*i *_is a feature vectors and *y*_*i *_corresponds to a class label. The boosting approach constructs a sequence of functions (also known as classifiers or hypotheses) *h*($\vec{x}$), indexed by the parameter *t*; given by an instance (*x*_*i*_, *y*). The *k*^*th *^component of *h*($\vec{x}$) reflects the confidence of the classifier that label *k *corresponds to the true class label *y*. The components of *h*($\vec{x}$) are normalized, so that *h*($\vec{x}$) specifies a distribution over labels. Associated to each instance(*x*_*i*_, *y*_*i*_) is a weight ${\text{w}}_{\text{i}}^{\text{t}}$, which, when normalized, yields a probability distribution {$P_{i}^{t}$, *i *= 1,... *N*} over the training data. This distribution is supplied to the "subroutine" that actually constructs the classifier associated with boosting round *t*. The "subroutine" then uses these weights to construct the classifier *h*($\vec{x}$). The error of the classifier constructed on round *t *are identified, and *h*($\vec{x}$) relative to the distribution supplied on round *t*, and {$P_{i}^{t}$} are calculated. The coefficient of the classifier *h*($\vec{x}$) in the combined hypothesis can then be calculated. The weights are then updated using the update rules. Those weights corresponding to instances *x *for which *h*($\vec{x}$) assigns a large probability mass (near 1) to the correct class are decreased substantially more than those for which *h*($\vec{x}$) assigns a small probability mass. It follows that the distribution $P_{i}^{t}$ becomes more concentrated on those instances that have a high rate of misclassification. We then classify an attribute vector *x *by computing the linear combinations of *h*($\vec{x}$). This procedure is combined with bagging.

Bagging with CBoost can reduce variance error but not affect bias error [10]; this algorithm averages the predictions of several classifiers and then assigns the class label that is closest to the average.

## Conclusion

We applied our IUP predictor to the problem of identifying both IUP and structured regions in proteins. We have developed a new ensemble method called bagging with CBoost; that have improved the overall performance of our IUP predictor. We find that both feature selection and ensemble methods improve the performance. Also we find that extracting features based on physiochemical proprieties proved beneficial. Augmenting features derived from physiochemical proprieties of amino acids (such as hydrophobicity, complexity, etc.) followed by and feature selection step, and developing bagging with CBoost algorithm significantly improve the predicting accuracies on both IUP and structured proteins. Those are the key innovations of this version of IUP predictor. Because performance of IUP is on par with PONDR, IUP is a viable alternative predictor that can be useful in structural genomics.

## Results

### Data

Both IUP and structured protein training data are selected from the Protein Data Bank (PDB) based on X-ray crystallography or other reliable experimental results. IUP residues are verified in PDB as missing coordinates in X-ray crystallography data. The selected structured protein sequences consist of 290 non-redundant sequences and are completely folded proteins with stable 3D structures, and resolutions better than 2A. Their pair-wise sequence identities are less than 25% (PDB SELECT25).

### Experimental results

Structural information on IUP and structured proteins for training are cross verified using PDB to ensure the reliability of training data. The test dataset are out of sample data.

It appears that the performance of the IUP predictor depends on several parameters, including the window length L for feature generation, the number of decision making instances K inside a RMCT tree node and the number of tree nodes N that are used for decisions. Experimental results indicate that N is least sensitive and L is most sensitive, therefore, we focused on a series of experiments using a variety of different window L lengths L and decision-making instances K.

We performed the experiments to test how the number of decision-making instances affects the performances of IUP prediction. As shown in Table 3, The classifier achieved the best performance in detecting IUP regions when the number of decision-making instances K equals to 9. However, the performance of identifying structured residues increases as K increases (monotonic increase function). To balance the predicting accuracies between IUP and structured residue simultaneously, we set K = 21 as default value in our predictor, as this value appears to offer best overall classification accuracy.

**Table 3:** The effect of decision-making instances (K) on the performance of IUP

K	TP (IUP)	TN (Order)	Average accuracy
3	0.8031	0.7183	0.7607
5	0.8014	0.7336	0.7675
7	0.804	0.7349	0.7694
9	0.8106	0.7414	0.776
11	0.8036	0.7453	0.7744
13	0.8015	0.749	0.7752
15	0.8023	0.7514	0.7768
17	0.8025	0.7536	0.778
19	0.8024	0.7575	0.7799
21	0.8005	0.7597	0.7801
23	0.7964	0.7624	0.7794

**Figure 2:**
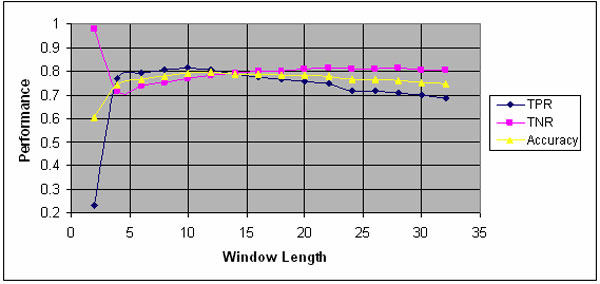
The effect of window length for feature extraction on the performance of IUP.

Window length L significantly affects the performance as showing in Figure 2. The performances on IUP prediction is marked in blue, while pink line is the performance on predicting structured residues. Yellow line represents the overall accuracy. In Figure 2, we set our default window length L = 10, as this value appears to give a best overall result. Default value for N is set at N = 3.

We made several observations, namely:

▪ When the window length for extracting feature is less than 4, the predictor almost cannot identify any IUP region. This implicitly suggests existence of local force on each residue, and this force determines the fold of protein. It also indicates that a structural or functional motif contains several or more amino acid residues; window length L should not be too short. It should not be too long as a long sequence may contain several different structural or functional domains or motifs.

▪ When window length L is approximately 10, the true positive rate reaches a maximum; beyond that, the true positive rate decreases as the window length increases.

▪ The average accuracy reaches a maximum when window length is approximately 12; beyond that, the average accuracy keeps decreasing.

▪ The true negative rate increases when window length L increases; after L is approximately increased to 28, the rate drops.

▪ The three rate curves (true positive, true negative, overall) are crossed approximately at window length 14 (See Figure 2).

**Figure 3:**
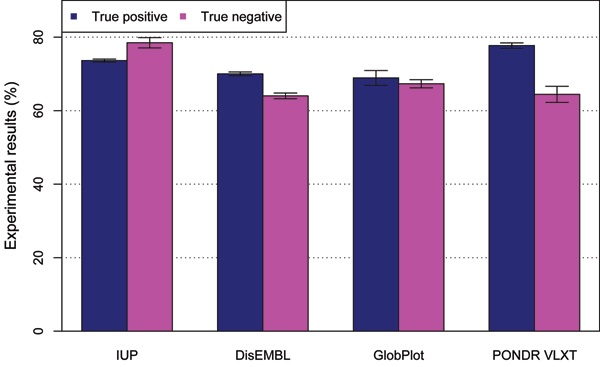
Comparison of our predictor (IUP) to DisEMBl, GlobPlot and PONDR VLXT.

We compared IUP performance against other predictors such as PONDR, GlobPlot and DisEMBL. Both GlobPlot and DisEMBL are developed by European Molecular Biology Laboratory (EMBL). While DisEMBL is based on the neural networks, GlobPlot is a single-feature (IUP propensity) classifier for IUP prediction. PONDR is predictor mainly based on neural network algorithm. In order to reliably test the performance of our IUP predictor, the test data was completely out-of-sample "blind" data and consist of 255 both IUP and ordered proteins. The prediction and comparison results are illustrated in Figure 3. The test result indicated the IUP predictor reached the same performance level of the most popular predictor PONDR and outperformed disEMB, and Globplot. For many test sequences, we found the IUP predictor performed best, while for many others, PONDR performed best. This suggested that our IUP predictor is a viable alternative to the most popular predictor – PONDR.

## Competing interests

The authors declare that they have no competing interests.

## Authors' contributions

Both authors contributed equally to the paper.
